# A standardized combination of *Sphaeranthus indicus* and *Mangifera indica* extracts improves antioxidant defense and anabolic signaling and attenuates dexamethasone-induced skeletal muscle atrophy

**DOI:** 10.29219/fnr.v70.14578

**Published:** 2026-07-17

**Authors:** Sreenath Kundimi, Thirupathi Rao, Geum Duck Park, Kyung Seok Kim, Soo Ro Kim, Krishnaraju Venkata Alluri, Krishanu Sengupta

**Affiliations:** 1 Laila Nutra Private Limited, JRD Tata Industrial Estate, Vijayawada, India; 2 Suheung Technology Research Institute, Gwacheon-si, Republic of Korea

**Keywords:** dexamethasone, *Mangifera indica*, oxidative stress, PI3K/AKT/mTOR signaling, skeletal muscle atrophy, *Sphaeranthus indicus*

## Abstract

**Background:**

Skeletal muscle atrophy is characterized by impaired protein synthesis, increased proteolysis, oxidative stress, and mitochondrial dysfunction. Phytoceutical interventions with antioxidant and cytoprotective properties may offer therapeutic potential in muscle-wasting conditions.

**Objective:**

To assess the effects of LI12542F6 SMI, a standardized formulation of *Sphaeranthus indicus* (SI) flower heads and *Mangifera indica* (MI) bark extracts (2:1), on dexamethasone (DEX)-induced skeletal muscle atrophy and to elucidate the underlying molecular mechanisms.

**Design:**

*In vitro* studies assessed antioxidant activity, endothelial nitrite production, protein synthesis, and the activation of the phosphatidylinositol 3-kinase/protein kinase B/mammalian target of rapamycin signaling (PI3K/AKT/mTOR) pathway, with or without DEX. *In vivo*, male Sprague Dawley rats received DEX (0.1 mg/kg body weight) and were supplemented with SMI (0, 45, or 90 mg/kg body weight) for 12 days. Outcomes included lean body mass, grip strength, muscle histology, antioxidant enzyme activity, and the expression of anabolic and catabolic protein markers.

**Results:**

SMI enhanced reactive oxygen species scavenging and increased endothelial nitrite production via PI3K/AKT-mediated activation of endothelial nitric oxide synthase. In DEX-treated rats, SMI attenuated declines in lean body mass, grip strength, and muscle fiber morphology. Mechanistically, SMI improved antioxidant markers, activated PI3K/AKT/mTOR signaling and myogenic markers, and suppressed catabolic and apoptotic proteins. The hepatic or renal biochemical parameters were unaltered.

**Discussion:**

These findings indicate that SMI mitigates glucocorticoid-induced muscle atrophy through coordinated modulation of oxidative stress, mitochondrial function, and anabolic signaling pathways. The activation of PI3K/AKT/mTOR signaling appears central to its effects, linking antioxidant activity with enhanced protein synthesis and the suppression of apoptotic signaling.

**Conclusions:**

SMI attenuates DEX-induced skeletal muscle atrophy and improves functional and molecular markers. These findings suggest further investigation and clinical substantiation of this phytoceutical as a possible intervention for muscle-wasting conditions.

## Popular scientific summary

- Gradual loss of muscle mass and function occurs due to chronic stress, advancing age, or the use of corticosteroids, which are associated with oxidative stress and reduced protein synthesis.
- A combination of *Sphaeranthus* indicus (SI) and *Mangifera indica* (MI) extracts, LI12542F6 (SMI), demonstrated antioxidant and muscle-protective activities.
- SMI supported muscle protein synthesis and reduced mitochondrial membrane depolarization *in vitro*.
- In dexamethasone-induced rats, SMI maintained muscle mass and strength, suggesting the possibility for managing muscle-wasting conditions.

Skeletal muscle atrophy is characterized by a progressive decline in muscle mass, strength, and function, contributing to frailty, falls, and loss of independence. It can arise from diverse conditions, including aging, immobilization, cachexia, malnutrition, and glucocorticoid exposure. Sarcopenia refers specifically to age-related muscle loss and is increasingly recognized as a geriatric syndrome with significant clinical and public health implications (1). It affects approximately 10–20% of individuals aged 60 or older, with a higher prevalence in older populations (2), and is now included in the International Classification of Diseases (ICD-10) (3). However, multiple forms of muscle atrophy share common underlying mechanisms, including oxidative stress, mitochondrial dysfunction, and dysregulated protein turnover.

At the molecular level, skeletal muscle atrophy results from an imbalance between protein synthesis and degradation pathways. Oxidative stress plays a central role in this process, as excessive production of reactive oxygen species (ROS) damages cellular macromolecules and disrupts intracellular signaling (4). Mitochondrial dysfunction is a characteristic feature of aging skeletal muscle, acting both as a source and target of ROS, thereby amplifying oxidative damage and impairing cellular bioenergetics (5). Elevated ROS levels activate transcription factors such as forkhead box O (FOXO) and nuclear factor-κB (NF-κB), which stimulate the expression of muscle-specific E3 ubiquitin ligases, including Atrogin-1 and muscle RING finger-1 (MuRF-1), and promote ubiquitin-proteasome-mediated protein degradation (6). In addition, chronic low-grade inflammation (inflammaging) contributes to muscle loss by inhibiting myogenic differentiation and suppressing insulin/insulin-like growth factor-1 (IGF-1) signaling pathways, which are critical for maintaining muscle mass (7).

Plant-derived bioactive metabolites with antioxidant and anti-inflammatory properties have attracted increasing attention as potential modulators of skeletal muscle metabolism. Polyphenols and flavonoids can reduce oxidative stress, improve mitochondrial function, and regulate signaling pathways involved in muscle protein turnover (8). Metabolites such as mangiferin, apigenin, and ginsenosides have demonstrated protective effects against muscle degeneration in experimental models by suppressing inflammatory signaling and restoring anabolic pathways (9–11).

An array of scientific evidence substantiates that *Sphaeranthus indicus* L. (Asteraceae) flower heads contain several bioactive metabolites, including sesquiterpene lactones such as 7-hydroxyfrullanolide and flavonoids structurally related to quercetin and apigenin, which have been reported to exhibit antioxidant and immunomodulatory activities (12). Experimental studies have shown that 7-hydroxyfrullanolide suppresses pro-inflammatory mediators by modulating NF-κB signaling and exhibits anti-inflammatory activity *in vivo* (13). The stem bark of *Mangifera indica* L. (Anacardiaceae) contains abundant polyphenolic metabolites, particularly the C-glucosyl xanthone mangiferin, which exhibits strong antioxidant, anti-inflammatory, and cytoprotective properties (9, 14). Mangiferin has also been reported to regulate mitochondrial function and redox homeostasis as well as to influence signaling pathways involved in skeletal muscle metabolism (8). Standardized extracts of *M. indica* bark have demonstrated antioxidant and immunomodulatory activities, including the inhibition of Tumor necrosis factor alpha (TNF-α)-induced NF-κB activation and the attenuation of inflammatory responses in experimental models (9, 15, 16). These pharmacological activities are mechanistically relevant to skeletal muscle wasting, where oxidative stress and inflammatory signaling accelerate proteolysis and impair anabolic recovery.

Recent randomized placebo-controlled trials have reported improvements in muscle strength, lean mass, and endurance following supplementation with a standardized botanical formulation containing *Sphaeranthus indicus* and *Mangifera indica* extracts (17, 18). However, the molecular mechanisms underlying these effects remain unclear. Given the central role of oxidative stress and dysregulated anabolic signaling in skeletal muscle atrophy, bioactive metabolites with antioxidant and cytoprotective properties may modulate pathways governing muscle protein turnover. The activation of the phosphatidylinositol 3-kinase/protein kinase B/mammalian target of rapamycin (PI3K/AKT/mTOR) signaling axis promotes protein synthesis while suppressing FOXO-mediated transcription of atrophy-related genes (19).

Based on this mechanistic rationale, we hypothesized that the standardized botanical formulation SMI may attenuate skeletal muscle atrophy by restoring redox balance and activating anabolic signaling pathways. This proof-of-concept study demonstrates that the botanical combination mitigates glucocorticoid-induced muscle atrophy by regulating redox homeostasis and the PI3K/AKT/mTOR signaling pathway. In this investigation, we employed complementary *in vitro* and *in vivo* models, enabling a comprehensive assessment of functional performance along with biochemical and molecular markers of muscle atrophy.

## Materials and methods

### Test item

**Fig. 1:**
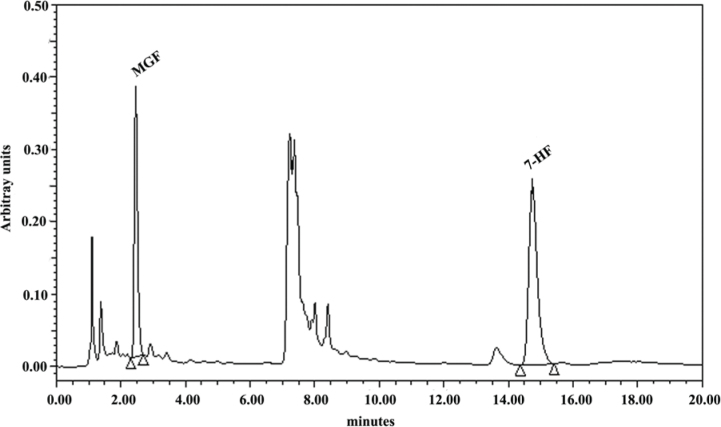
A typical HPLC chromatogram of SMI showing elution peaks of mangiferin (MGF) and 7-hydroxyfrullanolide (7-HF) at 210 nm with retention times of 2.461 and 14.744 min, respectively. HPLC: high-performance liquid chromatography.

LI12542F6 or SMI is a proprietary formulation containing a blend of *Sphaeranthus indicus* L. (Asteraceae) (SI) flower heads and *Mangifera indica* L. (Anacardiaceae) (MI) stem bark extracts (2:1). SI flower heads were collected from wild-crafted sources in Odisha, India, and MI bark was obtained from plantation-grown trees in Andhra Pradesh, India. A certified taxonomist authenticated the plant materials through morphological comparison with authenticated reference specimens. The voucher specimens of *S indicus* L. flower heads (voucher no. 6578) and *M indica* L. stem bark (voucher no. 6246) were deposited in the herbarium of the Taxonomy Division, Laila Nutra Private Limited, Vijayawada, India, for future reference. The dried plant materials were pulverized and extracted separately using ethanol-ethyl acetate for SI and aqueous ethanol for MI under controlled temperature conditions. The resulting extracts were individually filtered, concentrated under reduced pressure, and dried before combination. The resulting mixture was pulverized, sieved, and thoroughly mixed to obtain LI12542F6 as a uniform free-flowing powder. The formulation was standardized to contain not less than 4% 7-hydroxyfrullanolide (7-HF) and 2.5% mangiferin (MGF), as determined by high-performance liquid chromatography (HPLC). The phytochemical profile of LI12542F6 was analyzed using a HPLC system (Waters Acquity, Waters Corporation, Milford, MA, USA) equipped with an autosampler, a column oven with thermostatic control, a photodiode array detector, and a Empower 2 software. Samples were extracted with aqueous methanol and filtered through a 0.22 μm polyvinylidene difluoride (PVDF) membrane before analysis. Separation was performed on a Waters X Bridge C18 column (100 × 4.6 mm, 3.5 μm). The mobile phase consisted of solvent A (0.1% orthophosphoric acid in water) and solvent B (acetonitrile) at a flow rate of 1.0 mL/min. The gradient program started with 87% A and 13% B for 5 min, followed by a rapid change to 65% A and 35% B, which was maintained for 14 min. The column oven compartment was maintained at 40°C. MGF and 7-HF were detected at 210 nm with retention times of 2.461 and 14.744 min, respectively (Fig. 1), and were identified using authentic reference standards (Sigma-Aldrich, St. Louis, MO).

### Chemicals and reagents

The primary and secondary antibodies were procured from commercial sources (Supplementary Table S1). Cycloheximide (CHX, cat# 239763-M), dexamethasone (DEX, D4902), ethylenediaminetetraacetic acid (EDTA, cat# E6758), phenylmethylsulfonyl fluoride (PMSF, cat# 52332), sodium carboxymethyl cellulose (CMC-Na, cat# 419338), Lipopolysaccharides from *Escherichia coli* O111:B4 (LPS, cat# L2630), phorbol 12-myristate 13-acetate (PMA, cat# 52440), wortmannin (WORT, cat# 681675), rapamycin (RAPA, cat# R0395), 2’,7’-dichlorofluorescin diacetate (DCF-DA, cat# D6883), and other analytical-grade reagents were purchased from Sigma-Aldrich (St. Louis, MO).

### In vitro *experiments*

#### Cell culture and treatments

Primary human umbilical vein endothelial cells (HUVEC, cat# CRL-1730), Human leukemia (HL) promyelocytic cells HL-60 (cat# CCL-240), and L6 rat myoblast cells (cat# CRL-1458) were purchased from the American Type Culture Collection (ATCC, Manassas, VA). HUVECs were maintained in vascular cell basal medium supplemented with 10% fetal bovine serum (FBS, cat#16000044, Gibco, Waltham, MA), 1% penicillin-streptomycin, 4.5 g/L D-glucose, and 1 mM sodium pyruvate. HL-60 cells were cultured in RPMI medium (Hi-Media Labs, Mumbai, India) containing 10% FBS and 1% penicillin-streptomycin (HiMedia Laboratories Pvt. Ltd, Mumbai, India). L6 cells were maintained in Dulbecco’s Modified Eagle’s Medium (DMEM) supplemented with 10% FBS, 1% penicillin-streptomycin, 4.5 g/L D-glucose, and 1 mM sodium pyruvate. Cells were incubated at 37°C in a humidified atmosphere containing 5% CO_2_, and all experiments were conducted using mycoplasma-free cells between passages 5 and 10. All *in vitro* experiments were performed in three independent replicates, each conducted with duplicate culture wells.

For Western blot experiments, HUVECs were seeded at 3 × 10^5^ cells per well in 6-well plates and incubated at 37°C in 5% CO_2_ for 24 h. The following day, the medium was replaced with serum-free medium, and cells were maintained overnight before treatment. For L6 differentiation, cells at approximately 80% confluence were cultured in DMEM supplemented with 2% horse serum (cat# 16050122, Gibco, Waltham, MA) for eight consecutive days, with medium changes every 48 h. Fully differentiated myotubes were then serum-starved overnight. Serum-starved HUVECs or L6 myoblast cells were treated with SMI (1 μg/mL), with or without 10 μM WORT, for 30, 60, or 120 min for signaling studies. In a separate experiment, overnight serum-starved L6 myotubes were pre-treated with SMI (0.1 or 1.0 μg/mL) for 2 h, followed by DEX exposure for 72 h.

#### Cell viability assay

Cell viability was assessed using the Cell Counting Kit-8 (CCK-8, cat# 96992, Sigma-Aldrich, St. Louis, MO) to evaluate the cytotoxicity of the test samples. An equal number (10,000/well in a 96-well culture plate) of HL-60 cells in logarithmic growth were seeded in 100 μL of complete RPMI-1640 and maintained at 37°C in 5% CO_2_ overnight. Then, cells were treated with SI, MI, or SMI at different concentrations (0.5, 1.0, 2.5, 10, and 20 μg/mL) or vehicle control (0.1% DMSO, VC) and incubated for an additional 24 h. The wells containing medium without cells served as blanks. Following treatment, 10 μL of CCK-8 reagent was added directly to each well, and the plates were incubated for 2 h at 37°C in the dark. Absorbance was measured at 450 nm using a microplate reader (SpectraMax M5, Molecular Devices, San Jose, CA). Background-corrected absorbance values were used to calculate cell viability relative to VC using the formula:


$$\text{Cell viability }(\%)=\left[\left({\text{OD}}_{\text{treated}}-{\text{OD}}_{\text{blank}})/({\text{OD}}_{\text{control}}-{\text{OD}}_{\text{blank}}\right)\right]\times 100$$

Experiments were performed in triplicate wells and repeated independently three times.

#### DCF-DA assay

ROS levels in HL-60 cells were assessed using the DCF-DA assay (12). Cells (1.25 × 10^5^/well) were pre-incubated with DCF-DA for 30 min, then stimulated with 100 nM PMA, and immediately treated with 0.5–10 μg/mL of SI, MI, or SMI. Fluorescence was measured over 180 min (Ex: 460 nm / Em: 550 nm) using a multi-mode reader (Enspire, PerkinElmer, Shelton, CT).

#### Nitrite assay

Nitrite production in HUVEC cells was quantified using the Griess assay (20). Cells (0.5 × 10^6^/well) were seeded in 6-well plates and treated with SI, MI, or SMI (1–10 μg/mL) for 24 h. After treatment, 50 μL of conditioned media was mixed with 50 μL of freshly prepared Griess reagent [a 1:1 mixture of 1% sulfanilamide in 5% phosphoric acid and 0.1% N-(1-naphthyl)ethylenediamine dihydrochloride (NED) in distilled water] and incubated for 10 min at 37°C. Absorbance was measured at 550 nm using a Spectramax M5 microplate reader (Molecular Devices, San Jose, CA).

#### Western blot assay

Treated HUVEC, L6 myoblasts or myotubes, or gastrocnemius muscle (GM, 100 mg) were lysed in standard lysis buffer [10 mM Tris-HCl (pH 7.4) containing 1 mM EDTA, 150 mM NaCl, 1% (v/v) Triton X-100, 0.5% (w/v) sodium deoxycholate, 1 mM PMSF, 10 μg/mL aprotinin, 10 μg/mL leupeptin, 1 μM pepstatin, 1 mM sodium fluoride, and 1 mM sodium orthovanadate]. Lysates were clarified (13,000 × g, 15 min, 4°C), and protein contents were determined via bicinchoninic acid (BCA) assay (Thermo Scientific, Waltham, MA). Proteins were separated in SDS-PAGE, transferred to PVDF membranes, and probed with primary antibodies (overnight, 4°C), followed by HRP-conjugated secondary antibodies. Bands were visualized using chemiluminescence (ChemiDoc MP imaging system, Bio-Rad, Hercules, CA) and quantified using ImageJ (v1.53e; NIH, Bethesda, MD). Protein levels were normalized to unphosphorylated proteins or β-actin/Glyceraldehyde-3-phosphate dehydrogenase (GAPDH).

#### Protein synthesis assay

Protein synthesis in L6 myoblasts was quantified by L-homopropargylglycine (HPG) incorporation using the Click-iT™ HPG Alexa Fluor™ 488 assay kit (cat# C10428, Thermo Scientific, Waltham, MA) per manufacturer’s protocol. In 96-well culture plates, an equal number of cells (20,000/well) were maintained (37°C, 5% CO_2_) overnight. After replacing the medium, the cells were treated with SMI (0.1 or 1.0 μg/mL) prepared in a working stock solution of Click-iT HPG prepared in methionine-free RPMI (cat# A1451701-01, Gibco, Waltham, MA). The cells were treated with SMI in the presence or absence of WORT, RAPA, or CHX for 30 min. Fluorescence (Ex: 495 nm/Em: 519 nm) was measured using a microplate reader (Enspire, PerkinElmer, Shelton, CT).

#### Mitochondrial membrane potential (MMP, ∆ψm) assay

MMP (∆ψm) was assessed using 5,5,6,6’-tetrachloro-1,1’,3,3’-tetraethylbenzimidazylcarbocyanine iodide (JC-1, 2 μM; cat# T3168, Thermo Fisher Scientific, Waltham, MA), as described earlier (21). L6 myoblast cells (50,000/well) were seeded in a 6-well plate, incubated overnight, and then treated with DEX (25 μM) in the presence or absence of SMI (0.1 or 1.0 μg/mL) for 72 h. Following treatment, the cells were stained with JC-1 for 30 min in the dark. The stained cells were washed, trypsinized, and resuspended in a 2% fluorescence-activated cell sorting (FACS) buffer. Flow cytometric acquisition was performed using a BD FACSVerse flow cytometer (Franklin Lakes, NJ). The gating strategy involved first acquiring unstained control cells to establish the baseline population and set the quadrant gates. Subsequently, JC-1-stained control cells were acquired to adjust and confirm quadrant settings. DEX-treated cells, with or without LI12542F6, were then analyzed using the predefined quadrant gates. Mitochondrial membrane potential was expressed as the ratio of red to green fluorescence intensity.

### In vivo *study*

#### Animal husbandry and ethics approval

Male Sprague Dawley rats (10–12 weeks old; body weight (BW): 260–320 g) were obtained from the RCC Laboratories India Pvt. Ltd. (Hyderabad, India). The animals were housed in the animal facility of Laila Impex R&D Center, Vijayawada, India (License no. 204/PO/Rc/S/2000/CPCSEA) under identical environmental conditions (22 ± 3°C, relative humidity 30–70%, 12 h light/12 h dark cycle) with free access to standard laboratory chow and water throughout the study. All experimental procedures were conducted in accordance with the guidelines of the Committee for the Purpose of Control and Supervision of Experiments on Animals (CPCSEA), Government of India, and were approved by the Institutional Animal Ethics Committee (IAEC) of Laila Impex R&D Center, Vijayawada, Andhra Pradesh, India (protocol no. LI240509, dated May 25, 2024). All procedures involving animals were conducted in accordance with the ARRIVE 2.0 (Animal Research: Reporting of *In Vivo* Experiments) guidelines. This study was conducted between July 2024 and August 2024.

#### Experimental design

The efficacy of SMI was evaluated in a well-established model for studying glucocorticoid-induced muscle wasting (22, 23). A total of 40 rats were randomly assigned to four groups based on BW: G1 (Vehicle), G2 (DEX 0.1 mg/kg), G3 (DEX + SMI 45 mg/kg), and G4 (DEX + SMI 90 mg/kg). The sample size (*n* = 10 rats per group) was determined based on the group sizes used in previous pharmacological studies evaluating glucocorticoid-induced skeletal muscle atrophy and functional outcomes (22, 23). Dexamethasone (0.1 mg/kg BW/day, intraperitoneally) was administered from days 5 to 12 to induce skeletal muscle atrophy. The dose and treatment duration were selected based on established models of glucocorticoid-induced muscle wasting that reliably induce morphological and functional alterations in skeletal muscle (22, 23). The SMI doses were administered orally (days 1–12), selected based on the conversion of clinically used human doses to rat-equivalent doses using the body surface area-based method (18).

BWs were measured on days 1, 5, 8, and 12 on a weighing balance (GE7101, Sartorius AG, Göttingen, Germany). All animals completed the experimental protocol, and no animals or data points were excluded from the analyses, except for Western blot evaluation of muscle protein markers. Analyses were performed using a subset of samples (*n* = 6) from each group. Post-experiment, blood samples were collected *via* retro-orbital plexus puncture under isoflurane anesthesia. Rats were sacrificed (CO_2_ inhalation), and the GM and soleus muscle (SM) were excised, weighed on an analytical balance (CP224S, Sartorius AG, Göttingen, Germany), fixed for histology, or snap-frozen for biomarker analysis. All outcome assessments, including functional measurements, muscle histology quantification, densitometric analysis, immunoblots, and data analysis, were performed using coded samples to minimize observer bias.

#### Randomization, allocation concealment, and blinding

Animals were randomly assigned to experimental groups following baseline body weight assessment to ensure balanced group distribution. Group allocation was performed before treatment initiation, and treatment identity was concealed using coded cages and sample identifiers. Outcome assessments were conducted using coded samples to minimize observer bias. Investigators performing forelimb grip strength measurements, histological evaluations, densitometric analyses of immunoblots, and data analyses were blinded to treatment allocation. Histological sections were coded before image acquisition and before quantification of muscle fiber cross-sectional area. Blinding was maintained until completion of data collection and primary statistical analyses.

#### Measurement of forelimb grip strength and calf thickness

Forelimb grip strength was measured using a T-bar grip strength meter (Orchid Scientific, Mumbai, India). Each rat underwent three trials, and the average force (Newtons) was recorded (24). Calf thickness was measured bilaterally using a digital Vernier caliper (VWR, Leuven, Belgium).

#### Dual X-ray Absorptiometry (DEXA)

Lean body mass was assessed on the final day of the study using DEXA (25). Each rat was anesthetized and positioned supine on the scanning platform of a densitometer (QDR Discovery A, S/N 82382, Hologic Inc., Bedford, MA) with its limbs fully extended. Scans were conducted using the small animal application with rat-specific whole-body software (v13.4.2).

#### Muscle fiber morphometry

GM and SM were fixed in 10% neutral buffered formalin, processed, paraffin-embedded, sectioned (4–6 μm), and stained with Picro Sirius Red. Sections were imaged at 20× magnification (Axio Vision Observer Z1, Carl Zeiss, Thornwood, NY) using a ProgRes C5 CCD camera. Muscle fiber cross-sectional area (CSA) was quantified using ImageJ software (v1.53e; NIH, Bethesda, MD) by analyzing 50 randomly selected fibers per section (24).

#### Data and statistical analysis

Data are expressed as mean ± standard deviation (SD). Statistical analyses were performed using GraphPad Prism (version 5.0, GraphPad Software, San Diego, CA, USA). For *in vitro* experiments comparing two experimental conditions, statistical significance was evaluated using an unpaired Student’s *t*-test. Data normality was assessed using the Shapiro–Wilk test prior to conducting parametric analyses. For comparisons involving a single factor, one-way analysis of variance (ANOVA) followed by Dunnett’s post hoc test was applied; for experiments involving two factors, two-way ANOVA followed by Bonferroni’s multiple-comparison test was used. Thus, appropriate corrections for multiple comparisons were consistently applied as appropriate. A *P*-value < 0.05 was considered statistically significant. Original Western blot images are provided in the supplementary file. In SMI-treated rats, recovery percentage from DEX-induced loss of lean body mass, grip strength, and muscle fiber CSA was calculated using the following formula:


[(Treatment−DEX)/(Vehicle control−DEX)]×100

## Results

### SMI enhances ROS scavenging and nitric oxide signaling through PI3K/AKT/eNOS activation

#### ROS scavenging activity

**Fig. 2:**
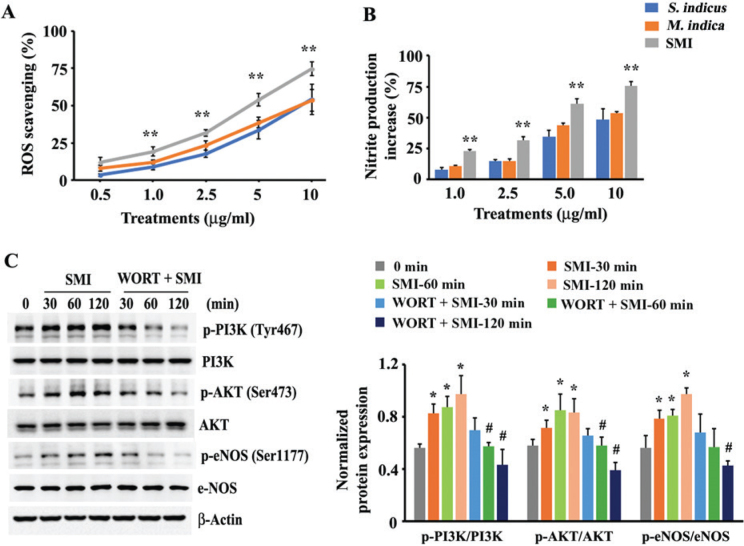
SMI enhances ROS scavenging, nitric oxide production, and PI3K/AKT/eNOS signaling. (A) ROS scavenging activity of *Sphaeranthus indicus* (SI), *Mangifera indica* (MI), and their combination (SMI) in PMA-stimulated HL-60 cells. (B) Nitrite production in HUVECs following treatment with SI, MI, or SMI. (C) Representative immunoblots and densitometric analysis showing phosphorylation of PI3K, AKT, and eNOS in HUVECs treated with SMI in the presence or absence of wortmannin (WORT). Data are presented as mean ± SD from three independent experiments. ROS: reactive oxygen species; PMA: phorbol 12-myristate 13-acetate; HUVEC: human umbilical vein endothelial cells; P13K/AKT: phosphatidylinositol 3-kinase/protein kinase B; eNOS: endothelial nitric oxide synthase. *P < 0.05 vs. control; **P < 0.05 SMI vs. SI or MI; ^#^*P* < 0.05 vs. SMI-treated group.

The effects of SI, MI, and their combination (SMI) on ROS generation were evaluated in PMA-stimulated HL-60 cells. Concentration-dependent reductions in ROS levels were observed (Fig. 2A). The mean IC_50_ values (± SD) for SI, MI, and SMI were 9.03 ± 1.71, 9.09 ± 1.69, and 4.06 ± 0.67 μg/mL, respectively. Across the concentration range of 1–10 μg/mL, SMI demonstrated significantly greater ROS scavenging activity than the individual extracts (*P* < 0.05). Consistent with these observations, the IC_50_ of SMI was significantly lower than that of SI (*P* = 0.0251) or MI (*P* = 0.0233).

Cell viability analysis indicated that treatment with SI, MI, or SMI at concentrations up to 20 μg/mL did not significantly affect HL-60 cell viability, suggesting that the observed antioxidant effects were not associated with cytotoxicity (Supplementary Fig. S1).

#### Nitrite production in endothelial cells

The effects of SI, MI, and SMI on nitric oxide (NO) production were examined in HUVECs by measuring nitrite levels. All treatments increased nitrite production in a concentration-dependent manner (Fig. 2B). At each concentration tested (1–10 μg/mL), SMI produced significantly higher nitrite levels than SI or MI alone (*P* < 0.05). At 10 μg/mL, SMI increased nitrite production by approximately 75.7%, whereas SI and MI increased nitrite levels by 48.5% (*P* = 0.0184) and 53.5% (*P* = 0.0033), respectively.

#### Activation of PI3K/AKT/eNOS signaling

To determine whether NO production was associated with endothelial signaling activation, phosphorylation of phosphatidylinositol 3-kinase (PI3K), protein kinase B (AKT), and endothelial nitric oxide synthase (eNOS) was assessed in HUVECs following SMI treatment. SMI induced a time-dependent increase in phosphorylation of all three proteins (Fig. 2C). Compared with untreated controls, phosphorylation of PI3K, AKT, and eNOS increased by 47.0% (*P* = 0.0112), 24.2% (*P* = 0.0356), and 40.0% (*P* = 0.0353), respectively, at 30 min; 55.3% (*P* = 0.0150), 46.3% (*P* = 0.0462), and 45.0% (*P* = 0.0283) at 60 min; and 72.7% (*P* = 0.0334), 42.9% (*P* = 0.0384), and 73.7% (*P* = 0.0071) at 120 min of treatment.

Cotreatment with the PI3K inhibitor wortmannin significantly attenuated SMI-induced phosphorylation in a time-dependent manner. Relative to SMI alone, wortmannin reduced phosphorylation of PI3K, AKT, and eNOS by 15.6% (*P* = 0.1293), 8.2% (*P* = 0.2488), and 13.4% (*P* = 0.3342) at 30 min; 34.5% (*P* = 0.0165), 30.1% (*P* = 0.0399), and 30.0% (*P* = 0.0866) at 60 min; and 55.4% (*P* = 0.0080), 52.7% (*P* = 0.0078), and 56.1% (*P* = 0.0002) at 120 min of treatment (Fig. 2C).

### SMI activates PI3K/AKT-dependent mTOR signaling and protein synthesis in L6 myoblast cells

**Fig. 3:**
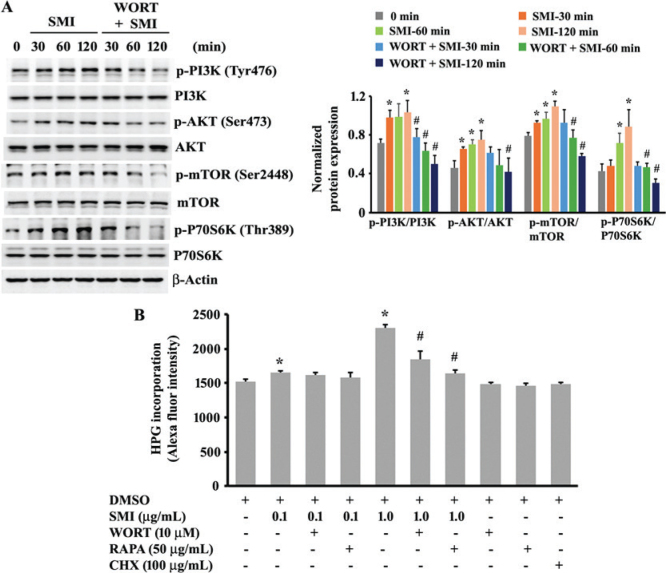
SMI activates PI3K/AKT/mTOR/P70S6K signaling and increases protein synthesis in L6 myoblasts. (A) Representative immunoblots and densitometric analysis of phosphorylated PI3K, AKT, mTOR, and P70S6K in L6 myoblasts treated with SMI with or without wortmannin (WORT). (B) Nascent protein synthesis assessed by HPG incorporation in cells treated with SMI alone or in combination with WORT, rapamycin (RAPA), or cycloheximide (CHX). Data are presented as mean ± SD from three independent experiments. P13K/AKT/mTOR: phosphatidylinositol 3-kinase/protein kinase B/mammalian target of rapamycin; HPG: L-homopropargylglycine. *P < 0.05 vs. vehicle control; ^#^*P* < 0.05 vs. SMI-treated group.

To investigate the molecular mechanisms underlying the anabolic effects of SMI in L6 myoblasts, we assessed the PI3K/AKT/mTOR signaling activation using immunoblot assays. SMI treatment increased p-PI3K levels by 37.3% (*P* = 0.0112), 38.5% (*P* = 0.0581), and 44.7% (*P* = 0.0332) at 30, 60, and 120 min, respectively. Similarly, p-AKT increased by 42.6% (*P* = 0.0352), 53.1% (*P* = 0.0139), and 62.9% (*P* = 0.0171) at these same time points (Fig. 3A). The downstream p-mTOR was increased by 17.0% (*P* = 0.0088), 21.8% (*P* = 0.0312), and 38.3% (*P* = 0.0020), while p-P70S6K increased by 12.8% (*P* = 0.3990), 68.7% (*P* = 0.0171), and 107.9% (*P* = 0.0300) at 30, 60, and 120 min, respectively (Fig. 3A).

Cotreatment with WORT attenuated SMI-induced phosphorylation of PI3K, AKT, mTOR, and P70S6K proteins (Fig. 3A). Compared to SMI alone, WORT reduced p-PI3K levels by 20.7% (*P* = 0.0379), 35.7% (*P* = 0.0240), and 51.6% (*P* = 0.0045), and p-AKT levels by 6.2% (*P* = 0.3601), 31.1% (*P* = 0.1356), and 43.5% (*P* = 0.0326) at 30, 60, and 120 min, respectively. The p-mTOR expressions were decreased by 20.6% (*P* = 0.0353) and 46.9% (*P* = 0.0006), and p-P70S6K by 35.4% (*P* = 0.0304) and 65.9% (*P* = 0.0237) at 60 and 120 min, respectively, while changes at 30 min were minimal and not significant (Fig. 3A).

The Click-iT HPG assay (Fig. 3B) demonstrated that SMI enhanced *de novo* protein synthesis in L6 myoblasts, increasing incorporation by 8.8% (*P* = 0.0134) and 51.7% (*P* < 0.0001) at 0.1 and 1.0 μg/mL, respectively. Cotreatment with WORT or RAPA attenuated the SMI-induced protein synthesis at 1.0 μg/mL, reducing incorporation by 19.9% (*P* = 0.0138) and 28.4% (*P* < 0.0001), respectively. In contrast, at 0.1 μg/mL SMI treatment, the inhibitory effects of WORT and RAPA were minimal and not statistically significant (Fig. 3B).

### SMI modulates myogenic proteins and attenuates apoptosis and atrophy-related marker proteins in DEX-treated L6 myotubes

**Fig. 4:**
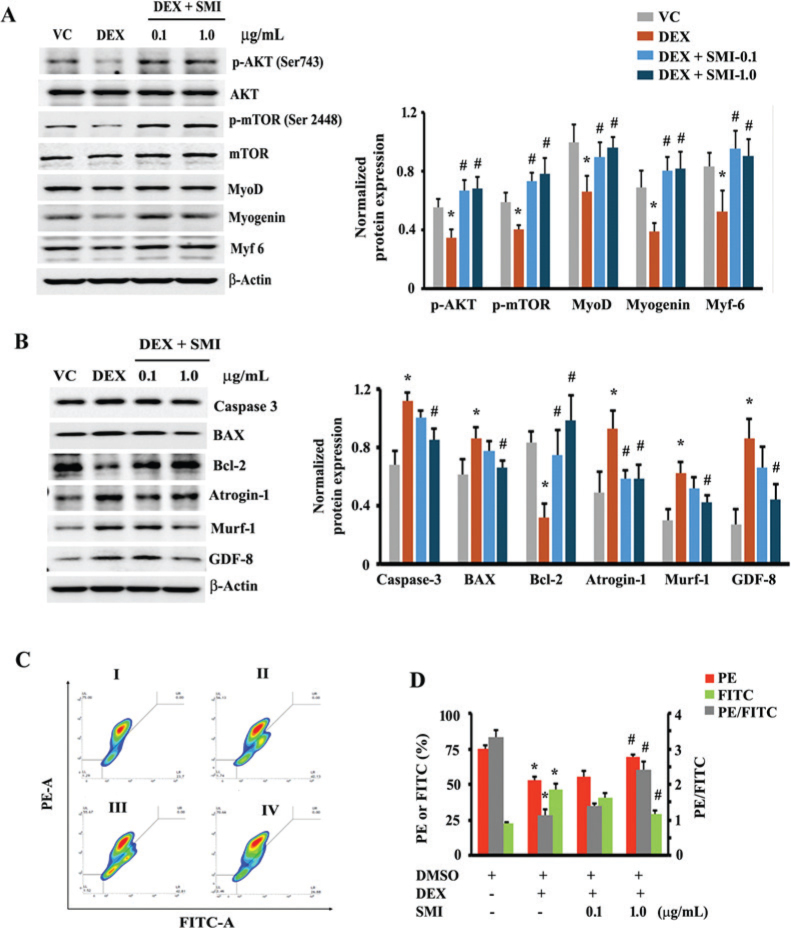
Effects of SMI on anabolic, apoptotic, and atrophy-related markers and mitochondrial membrane potential in DEX-treated L6 cells. (A) Representative immunoblots and densitometric analysis of p-AKT, p-mTOR, and myogenic regulatory proteins (MyoD, Myogenin, and Myf-6) in dexamethasone (DEX)-treated L6 myotubes with or without SMI. (B) Expression of apoptotic proteins (Caspase-3, Bax, and Bcl-2) and atrophy-related markers (Atrogin-1, MuRF-1, and GDF-8). (C) Representative JC-1 flow cytometry plots showing mitochondrial membrane potential. I–IV represent vehicle control (VC), DEX, DEX + 0.1 μg/mL SMI, and DEX + 1.0 μg/mL SMI, respectively. (D) Quantification of JC-1 fluorescence ratios. Data are presented as mean ± SD from three independent experiments. DEX: dexamethasone; mTOR: mammalian target of rapamycin. *P < 0.05 vs. vehicle control; ^#^*P* < 0.05 vs. DEX-treated group.

In L6 myotubes, DEX reduced p-AKT and p-mTOR expressions by 38.2% (*P* = 0.0106) and 31.1% (*P* = 0.0325), respectively, as compared with VC. DEX also decreased the expression of key myogenic regulatory proteins MyoD, Myogenin, and Myf-6 by 33.2% (*P* = 0.0251), 44.1% (*P* = 0.0247), and 36.9% (*P* = 0.0433), respectively (Fig. 4A). At 0.1 μg/mL, SMI cotreatment recovered the DEX-induced loss of protein levels and elevated p-AKT, p-mTOR, MyoD, Myogenin, and Myf-6 levels by 94.2% (*P* = 0.0046), 80.9% (*P* = 0.0029), 35.6% (*P* = 0.0472), 107.9% (*P* = 0.0048), and 80.7% (*P* = 0.0182), respectively, from DEX treatment. Similarly, the higher dose of SMI (1.0 μg/mL) produced similar or greater effects, increasing p-AKT, p-mTOR, MyoD, Myogenin, and Myf by 97.7% (*P* = 0.0052), 93.5% (*P* = 0.0184), 44.7% (*P* = 0.0210), 111.6% (*P* = 0.0110), and 71.7% (*P* = 0.0248), respectively, as compared with DEX (Fig. 4A).

DEX-treated L6 myotubes showed significant elevation in caspase-3 and Bax levels by 63.7% (*P* = 0.0040) and 40.8% (*P* = 0.0361), respectively, and reduction in Bcl-2 levels by 62.3% (*P* = 0.0022), compared with VC. DEX also increased Atrogin-1, MuRF-1, and Growth differentiation factor-8 (GDF-8) expressions by 90.1% (*P* = 0.0168), 104.0% (*P* = 0.0066), and 213.0% (*P* = 0.0045), respectively, *vs*. VC. At 0.1 μg/mL, SMI decreased caspase-3 and Bax levels by 10.3% (*P* = 0.0583) and 10.5% (*P* = 0.1883), respectively, compared with DEX, while increasing Bcl-2 expression by 136.6% (*P* = 0.0293). Simultaneously, Atrogin-1, Murf-1, and GDF-8 expressions were also decreased by 36.3% (*P* = 0.0309), 16.2% (*P* = 0.1825), and 22.9% (*P* = 0.1586), respectively. The higher dose (1.0 μg/mL) yielded stronger protections, reducing caspase-3, Bax, Atrogin-1, Murf-1, and GDF-8 by 24.1% (*P* = 0.0111), 23.6% (*P* = 0.0217), 37.2% (*P* = 0.0236), 31.0% (*P* = 0.0393), and 48.6% (*P* = 0.0141), respectively, while increasing Bcl-2 by 214.5% (*P* = 0.0081) *vs*. DEX (Fig. 4B).

### SMI protects against DEX-induced mitochondrial membrane depolarization in L6 myoblasts

Flow cytometry analysis revealed that DEX treatment significantly increased (*P* < 0.002) the JC-1 green fluorescence, as represented by the mean red/green fluorescence ratio (1.14), as compared to the VC (3.32), indicating an increase in the monomeric form of JC-1 (membrane depolarization) through the disruption of MMP (Fig. 4C & 4D). Cotreatment with 1.0 μg/mL SMI attenuated (*P* < 0.005 *vs*. DEX) the membrane depolarization, as indicated by a mean red/green fluorescence ratio of 2.42, with increased red fluorescence (JC-1 aggregates) indicating alleviation from DEX-induced membrane depolarization (Fig. 4C & 4D).

### SMI mitigates DEX-induced loss of muscle mass and function in Sprague Dawley rats

**Fig. 5:**
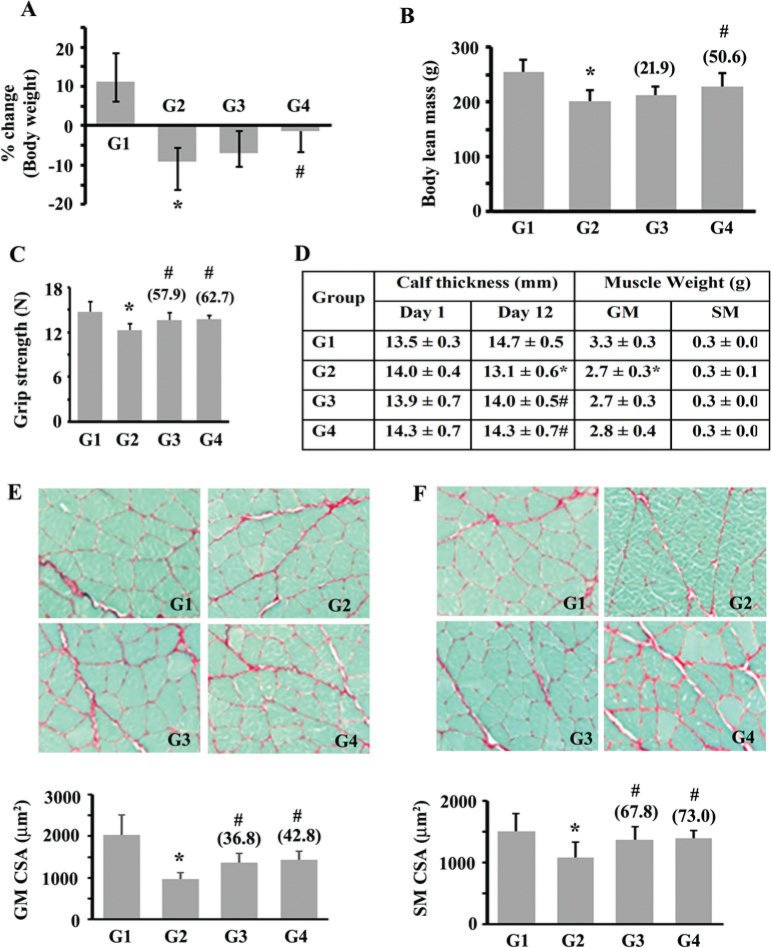
SMI attenuates DEX-induced reductions in lean body mass, muscle strength, and muscle fiber thickness in rats. (A) Body weight change, (B) lean body mass, and (C) forelimb grip strength in vehicle control (G1), DEX-treated (G2), and DEX + SMI-treated groups (G3 and G4). (D) Calf thickness, gastrocnemius muscle weight, and soleus muscle weight. Representative histological sections and quantification of muscle fiber cross-sectional area (CSA) in gastrocnemius (E) and soleus (F) muscles are shown. Data are presented as mean ± SD (*n* = 10). DEX: dexamethasone. *P < 0.05 vs. G1; ^#^*P* < 0.05 vs. G2.

DEX treatment (G2) significantly reduced (*P* < 0.05) BW, lean mass, grip strength, calf thickness, and GM weight compared to controls (G1) (Fig. 5A–D). SMI (45 and 90 mg/kg in G3 and G4, respectively) dose-dependently mitigated these effects, with 90 mg/kg showing significant (*P* < 0.05) recovery in BW (41.3%) (Fig. 5A), lean mass (50.6%) (Fig. 5B), grip strength (62.7%) (Fig. 5C), and calf thickness (75%) (Fig. 5D). Trends in recovery of GM and SM muscle weight were observed in the SMI-supplemented groups, but the differences were not significant (*vs.* G2) (Fig. 5D).

Morphometric analysis revealed that DEX-induced reduction in CSA (GM: 52.5%, SM: 28.4%) was recovered by SMI supplementation (*P* < 0.05 *vs*. G2) in GM (36.8 and 42.8% in G3 and G4, respectively) and SM (67.8 and 73% in G3 and G4, respectively) (Fig. 5E and 5F lower panels, respectively).

### SMI improves anabolic signaling and suppresses proteolytic and apoptotic pathways, and regulatory proteins in DEX-induced rats

**Fig. 6:**
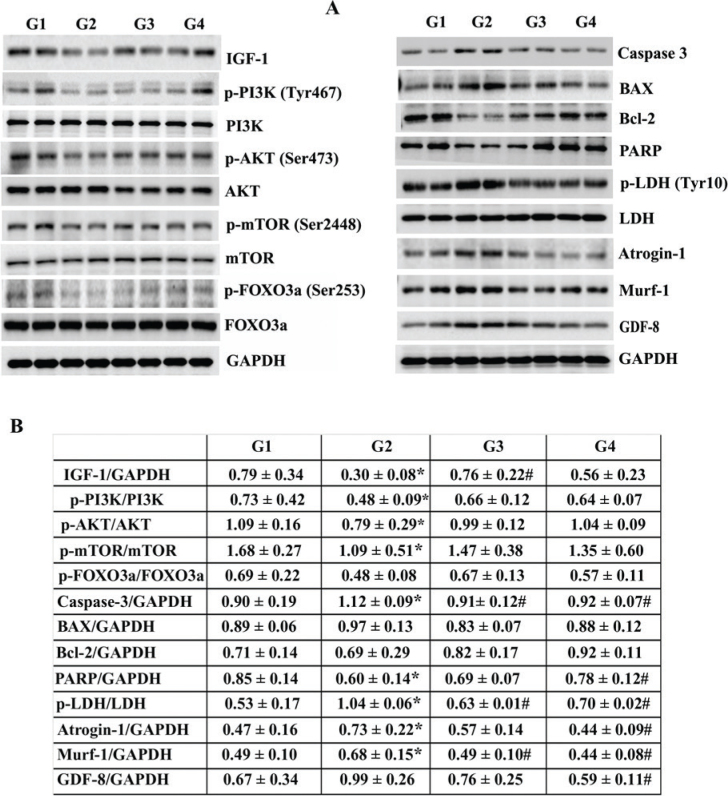
Effects of SMI on anabolic, catabolic, and apoptotic protein markers in the gastrocnemius muscle of DEX-induced rats. (A) Representative immunoblots showing proteins involved in anabolic signaling, proteolysis, and apoptosis, in gastrocnemius muscle from the vehicle control (G1), DEX-treated (G2), and DEX + SMI-treated groups (G3 and G4). (B) Densitometric quantification of normalized protein expression. Data are presented as mean ± SD (*n* = 6). DEX: dexamethasone. *P < 0.05 vs. G1; ^#^*P* < 0.05 vs. G2.

Figure 6A presents representative immunoblots showing the modulation of anabolic and catabolic protein expressions in the GM of the experimental rats. The table summarizes the group-wise means ± SD of normalized protein expression (relative to unphosphorylated proteins or GAPDH) obtained from densitometry analysis (Fig. 6B). DEX administration (G2) reduced Poly ADP Ribose Polymerase (PARP), p-mTOR, Bcl-2, IGF-1, and p-FOXO3a protein expressions and concurrently increased Caspase-3, BAX, Atrogin-1, Murf-1, and p-LDH protein expressions in the GM, compared with the vehicle control group (G1). SMI supplementation at 45 and 90 mg/kg BW (G3 and G4) improved these parameters. Atrogin-1, Murf-1, and GDF-8 protein levels were significantly reduced (*P* < 0.05 *vs*. G2) (Fig. 6B).

### SMI increases antioxidant defense and reduces muscle damage in DEX-induced rats

DEX (G2) reduced glutathione (GSH) levels (19.7%, *P* < 0.05) and suppressed glutathione peroxidase (GPx) (34.9%, not significant, NS) and CAT (27.7%, NS) activities in the GM, as compared to control rats (G1). Treatment with SMI, particularly at the 90 mg/kg BW dose, recovered muscle GSH (*P* < 0.05). However, the partially recovered GPx and CAT activities were not significant (*vs*. G2) (Table 1).

**Table 1:** Oxidative stress and biochemical markers in SMI-treated DEX-induced rats

Parameters	Vehicle control (G1)	DEX (G2)	DEX + SMI (45 mg/kg BW) (G3)	DEX + SMI (90 mg/kg BW) (G4)
Gastrocnemius muscle				
GPx (nmol/min/mg)	10.20 ± 2.38	6.64 ± 2.55	8.15 ± 1.15	9.24 ± 3.46
GSH (μM/mg)	5.24 ± 0.71	4.21 ± 0.98^*^	4.52 ± 0.28	5.33 ± 0.61^#^
CAT (μM/mg)	8.84 ± 2.13	6.39 ± 2.16	8.27 ± 1.22	7.79 ± 3.14
Serum				
BUN (mg/dL)	20.74 ± 2.43	21.10 ± 4.86	20.53 ± 3.01	20.93 ± 2.35
Creatinine (mg/dL)	0.29 ± 0.02	0.26 ± 0.03	0.26 ± 0.02	0.29 ± 0.06
AST (U/L)	175.2 ± 27.39	155.2 ± 24.85	188.7 ± 29.47	184.8 ± 39.51
ALT (U/L)	101.9 ± 23.28	104.6 ± 19.29	126.6 ± 30.56	117.0 ± 35.23
CK (U/L)	31.30 ± 14.38	56.40 ± 19.60^#^	41.96 ± 15.53	39.55 ± 12.61

Values are present as mean ± SD; *n* = 10.

* *P* < 0.05 vs. G1;

# *P* < 0.05 vs. G2 (one-way ANOVA followed by Dunnett’s post-hoc test).

ALT: Alanine aminotransferase; AST: Aspartate transaminase; BUN: Blood urea nitrogen; CAT: Catalase; CK: Creatine kinase; DEX: Dexamethasone; GSH: Glutathione; GPx: Glutathione peroxidase.

Serum biochemical parameters – blood urea nitrogen (BUN), creatinine, aspartate transaminase (AST), and alanine aminotransferase (ALT) – remained unaltered across all experimental groups, indicating no hepatic or renal toxicity. Serum creatine kinase (CK) levels were elevated by DEX (80.2% *vs*. G1, *P* < 0.05), while SMI treatment showed 25.6 (NS) and 29.9% (NS) reductions in the low (G3) and high (G4) dose groups, as compared to the G2 rats, respectively (Table 1).

## Discussion

Muscle atrophy involves progressive loss of skeletal muscle mass, integrity, and strength, arising from aging, glucocorticoid use, or chronic disease. Oxidative stress plays a central role in its pathogenesis, with elevated ROS levels promoting proteolysis via the activation of the ubiquitin–proteasome system, and upregulation of E3 ligases such as MuRF-1 and MAFbx (26, 27). Accordingly, targeting oxidative stress represents a rational strategy to mitigate muscle degeneration.

In this context, complementary *in vitro* models were used to investigate the mechanistic effects of SMI. HUVECs assessed eNOS activation and NO signaling, which are relevant to vascular and anabolic functions (28), and HL-60 cells evaluated the antioxidant properties of SMI (29). L6 myoblasts were employed to examine anabolic signaling, mitochondrial function, and myogenic differentiation (30). Together, these models enabled an integrated evaluation of SMI effects on redox balance, endothelial signaling, and muscle anabolic pathways.

SMI demonstrated greater ROS scavenging ability and NO production than the individual extracts, suggesting complementary activity among the constituent botanicals. Such interactions are consistent with the Loewe additivity model for combination effects (31). Increases in endogenous antioxidant markers, including GSH and GPx, activities in gastrocnemius muscle, consistent with the modulation of oxidative stress-related processes, accompanied the antioxidant activity.

NO is a key regulator of skeletal muscle physiology, influencing protein synthesis and signaling pathways, involving AMPK, PGC-1α, and PI3K/AKT/mTOR (32). In this study, SMI increased nitrite production and enhanced phosphorylation of PI3K, AKT, and eNOS, indicating the activation of NO signaling. AKT-mediated phosphorylation of eNOS at Ser1177 is critical for NO production (33), and wortmannin-mediated attenuation confirms the involvement of the PI3K/AKT pathway. These findings are consistent with evidence that eNOS activity is required for muscle regeneration (34).

Beyond endothelial signaling, SMI activated mTOR signaling, a central regulator of protein translation and muscle hypertrophy (32). The formulation contains bioactive polyphenols, including apigenin, quercetin, and mangiferin, which are associated with enhanced mitochondrial function and anabolic signaling (8, 35). Consistently, SMI increased protein synthesis by activating the PI3K/AKT/mTOR pathway. Although wortmannin and rapamycin attenuated this response, incomplete inhibition suggests involvement of additional regulatory pathways (36).

Upregulation of myogenic regulatory factors, including MyoD, myogenin, and Myf-6, supports a role for SMI in promoting muscle regeneration, as these transcription factors control satellite cell differentiation and repair (37, 38). SMI partially attenuated mitochondrial membrane potential in DEX-treated myoblasts, indicating improved mitochondrial function. Given that mitochondrial depolarization is an early trigger of catabolic signaling pathways, including AMPK-FOXO3, autophagy, and ubiquitin-proteasome-mediated proteolysis (39), the preservation of mitochondrial membrane potential may help limit cellular stress responses associated with muscle atrophy.

Consistent with these cellular findings, DEX administration in rats reduced IGF-1 expression and was accompanied by the suppression of anabolic signaling markers. SMI supplementation improved IGF-1 levels and was associated with increased phosphorylation of proteins within the PI3K/AKT/mTOR signaling pathway. IGF-1 is a key regulator of skeletal muscle growth and coordinates PI3K/AKT/GSK3β and PI3K/AKT/mTOR signaling to promote protein synthesis while suppressing FOXO-mediated transcription of atrophy-related genes (40).

SMI improved lean body mass, grip strength, and muscle fiber cross-sectional area in DEX-treated rats, consistent with enhanced anabolic signaling and reduced proteolysis at the molecular level. Grip strength is a validated indicator of muscle function and a key diagnostic criterion for sarcopenia (41, 42). Comparable protective effects have been reported for phytochemicals such as ginsenoside Rc and coumestrol, which attenuate glucocorticoid-induced muscle wasting by modulating protein degradation pathways (22, 23). SMI reduced the expression of key atrophy-associated proteins, including Atrogin-1, MuRF-1, and myostatin, which regulate the ubiquitin-proteasome pathway (43). It also modulated apoptotic signaling by increasing Bcl-2 and decreasing Bax and caspase-3 levels, consistent with reported effects of mangiferin on the Bcl-2/Bax axis (44). Reduced serum creatine kinase further indicates protection against muscle damage, while unchanged hepatic and renal biochemical parameters support a favorable signal safety profile *in vivo*.

SMI also influenced skeletal muscle energy metabolism. DEX increased lactate dehydrogenase phosphorylation, indicating a shift toward glycolysis and impaired mitochondrial oxidative capacity (45). SMI reduced this effect, suggesting partial restoration of oxidative metabolism. This may reflect its antioxidant properties and the activation of PI3K/AKT/mTOR signaling, both of which support mitochondrial function and metabolic homeostasis (46).

Although the overall outcome from the present study is a proof-of-concept, which is interesting and encouraging, this study has some limitations. First, the findings were generated using DEX-induced cellular and animal models, which may not fully represent the complex pathophysiology of age-related sarcopenia or other chronic muscle-wasting conditions. Second, although SMI treatment was associated with the activation of PI3K/AKT/mTOR signaling, the contribution of this pathway to the observed anti-atrophic effects was not directly evaluated using pathway inhibitors to confirm the effects. Third, mitochondrial assessment was limited to measuring mitochondrial membrane potential with JC-1 staining and therefore does not provide a comprehensive assessment of SMI’s effects on mitochondrial function. Further studies are warranted to clarify the underlying mechanisms and to evaluate the effects of SMI in long-term experimental models of muscle atrophy.

Taken together, the findings indicate that SMI treatment modulated oxidative stress, reduced mitochondrial membrane depolarization, and altered markers of anabolic and catabolic signaling pathways in experimental models of glucocorticoid-induced muscle atrophy. These observations provide a basis for further investigation of the mechanisms underlying the effects of this botanical formulation.

## Conclusion

**Fig. 7:**
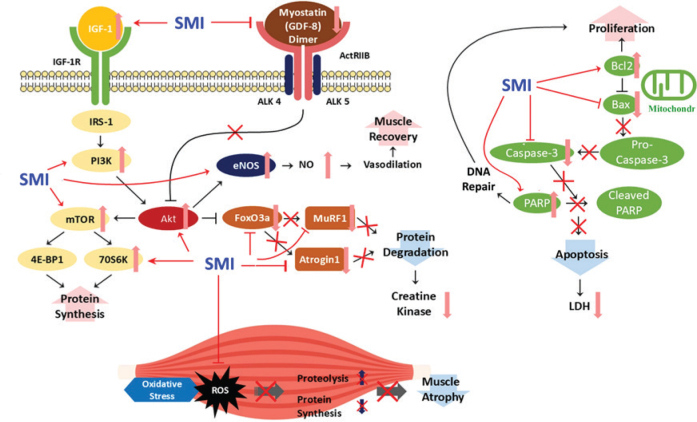
Proposed mechanisms of SMI in dexamethasone-induced skeletal muscle atrophy. SMI activates IGF-1/PI3K/AKT signaling, leading to mTOR activation and increased protein synthesis. It is also associated with inhibition of FOXO3a-mediated expression of Atrogin-1 and MuRF-1, reducing proteolysis. Additionally, SMI modulates eNOS activity, oxidative stress, mitochondrial function, and apoptotic markers (Bcl-2, Bax, caspase-3, and PARP). These combined effects are associated with attenuation of muscle atrophy. IGF-1: insulin-like growth factor-1; P13K: phosphatidylinositol 3-kinase; eNOS: endothelial nitric oxide synthase; mTOR: mammalian target of rapamycin.

The present study demonstrates that a standardized botanical formulation, SMI, comprising *Sphaeranthus indicus* flower heads and *Mangifera indica* bark extracts, attenuates DEX-induced skeletal muscle atrophy through multiple complementary mechanisms (Fig. 7). SMI reduced oxidative stress, improved mitochondrial membrane potential, and was associated with the modulation of protein expressions of anabolic pathways, including increased phosphorylation of components of the PI3K/AKT/mTOR signaling. These molecular changes were accompanied by improvements in lean body mass, grip strength, and muscle fiber cross-sectional area in DEX-treated rats. As a proof-of-concept study, these findings support further investigation to clarify the effects of this botanical formulation in longer-duration studies and to determine the translational relevance of these observations in clinical studies.

## Supplementary Material



## Data Availability

The data supporting the findings of this study are available within the article and its Supplementary file. Additional raw data are available from the corresponding author upon reasonable request.
