# Supplementary material for: A standardized combination of Sphaeranthus indicus and Mangifera indica extracts improves antioxidant defense and anabolic signaling and attenuates dexamethasone-induced skeletal muscle atrophy
Source: Food Nutr Res. 2026 Jul 17;70:10.29219/fnr.v70.14578. doi: 10.29219/fnr.v70.14578 (PMC13452269; doi:10.29219/fnr.v70.14578)
Supplement: Supplementary file 1 [file FNR-70-14578-s1.docx]

**Table S1: Primary and secondary antibody details**

| **S. No.** | **Antibody** | **Cat. No.** | **Type** | **Reactivity** | **Mol Weight (kDa)** | **Source** | **Manufacturer** | **Dilution** |
| --- | --- | --- | --- | --- | --- | --- | --- | --- |
| 1 | Phospho-mTOR (Ser2448) | 2971S | Primary | H M R Mk | 289 | Rabbit Polyclonal | Cell Signaling Technology, Danvers, MA | 1:1000 |
| 2 | mTOR | 2983S | Primary | H M R Mk | 289 | Rabbit Monoclonal |  | 1:1000 |
| 3 | Phospho-AKT (Ser473) | 9271S | Primary | H M R Hm Mk B Dg | 60 | Rabbit Polyclonal |  | 1:1000 |
| 4 | AKT | 9272S | Primary | H M R Hm Mk C B Dg Pg GP | 60 | Rabbit |  | 1:1000 |
| 5 | Phospho-eNOS (Ser1177) | 9571S | Primary | H B Pg | 140 | Rabbit |  | 1:1000 |
| 6 | eNOS | 9572S | Primary | H B Pg | 140 | Rabbit |  | 1:1000 |
| 7 | Phospho-FOXO3a (Ser253) | 9466S | Primary | H M R | 97 | Rabbit |  | 1:1000 |
| 8 | FOXO3a | 2497S | Primary | H M R Mk | 82 to 97 | Rabbit IgG |  | 1:1000 |
| 9 | BAX | 2772S | Primary | H M R Mk | 20 | Rabbit |  | 1:1000 |
| 10 | Bcl-2 | 2876S | Primary | H M R | 28 | Rabbit |  | 1:1000 |
| 11 | Caspase-3 | 9662S | Primary | H M R Mk | 17, 19, 35 | Rabbit |  | 1:1000 |
| 12 | PARP | 9542S | Primary | H M R Mk | 89, 116 | Rabbit |  | 1:1000 |
| 13 | Phospho-p70 S6 Kinase (Thr389) | 9205S | Primary | H M R Mk | 70, 85 | Rabbit |  | 1:1000 |
| 14 | p70S6 Kinase | 2708S | Primary | H | 70, 85 | Rabbit IgG |  | 1:1000 |
| 15 | Phospho-PI3 kinase p85 alpha/gamma (Tyr467, Tyr199) | BS-3332R | Primary | H R | 120 | Rabbit Polyclonal | Bioss Antibodies Woburn, MA | 1:1000 |
| 16 | PI3K | BSM-33219 | Primary | H M R C | 80 | Mouse Monoclonal |  | 1:1000 |
| 17 | Phospho-LDHA (Tyr10) | PA5-105445 | Primary | H | 37 | Rabbit Polyclonal | Thermo Fisher Scientific, Altham, MA | 1:1000 |
| 18 | LDH | PA5-27406 | Primary | H M R | 37 | Rabbit Polyclonal |  | 1:1000 |
| 19 | MyoD | MAB3878 | Primary | Ch, H, M, R | 45 | Mouse Monoclonal | Merck Millipore, Darmstadt, Germany | 1:1000 |
| 20 | Myogenin | MAB3876 | Primary | H M R | 34 | Mouse Monoclonal |  | 1:1000 |
| 21 | Myf-6 | SC-301 | Primary | H M R | 30 | Rabbit Polyclonal | Santa Cruz Biotechnology, Santa Cruz, CA | 1:1000 |
| 22 | Atrogin-1/ ATR-1 | 3535-100 | Primary | H M R | 43 | Rabbit Polyclonal | Bio-vision, Milpitas, CA | 1:1000 |
| 23 | Murf-1/TRIM63 | AF5366 | Primary | H M R | 41 | Goat Polyclonal | R&D systems, Minneapolis, MN | 1:1000 |
| 24 | IGF-1 | 28530-1-AP | Primary | H M R C | 7.7 | Rabbit Polyclonal | Protein Tech, San Diego, CA | 1:500 |
| 25 | GDF8/Myostatin | 19142-1-ap | Primary | H M R | 26 | Rabbit Polyclonal |  | 1:1000 |
| 26 | Anti- β-Actin Antibody | A2228-100UL | Primary | H M R | 42 | Mouse Monoclonal | Sigma-Aldrich, St. Louis, MO | 1:10000 |
| 27 | Peroxidase Affinipure Mouse anti-goat igG(H+L) | 205-035-108 | Secondary | H M Rb | NA | Anti-Goat | Jackson Immuno Research Laboratories, West Grove, PA | 1:10000 |
| 28 | Peroxidase Affinipure goat anti-rabbit igG(H+L) | 111-035-045 | Secondary | H | NA | Anti-Rabbit |  | 1:10000 |
| 29 | Peroxidase AffiniPure Goat Anti-Mouse IgG (H+L) | 115-035-062 | Secondary | H B Hr | NA | Anti-Mouse |  | 1:10000 |

Species Cross-Reactivity Key: H-Human, M-Mouse, R-Rat, Mk-Monkey, C-Chicken, B-Bovine, Dg-Dog, Pg-Pig, GP-Guinea pig, Hr-Horse

**Figure S1**

**Figure S1. Effects of S. indicus, M. indica, and SMI on HL-60 cell viability.** An equal number of HL-60 cells was treated with SI, MI, or SMI at concentrations ranging from 0.5 to 20 µg/mL for 24 hrs, as described in the Materials and Methods. Cell viability was assessed using the CCK-8 assay, and each bar represents the percentage of viable cells relative to vehicle-treated control (VC) cells. Data represent mean ± SD from three independent experiments, each performed in triplicate. Between-group comparison revealed no significant changes using an unpaired t-test.

Fig 2C

Fig 2C

Fig 2C

Fig 3A

Fig 3A

Fig3A

Fig 4A

Fig 4A

Fig 4A

Fig 4B

Fig 4B

Fig 4B

Fig 6A

Fig 6A

Fig 6A

Fig 6A

Fig 6A

Fig 6A

Fig 6A

Fig 6B

Fig 6B

Fig 6B

Fig 6B
